# Systematic Identification and Characterization of Novel Human Skin-Associated Genes Encoding Membrane and Secreted Proteins

**DOI:** 10.1371/journal.pone.0063949

**Published:** 2013-06-20

**Authors:** Peter Arne Gerber, Peter Hevezi, Bettina Alexandra Buhren, Cynthia Martinez, Holger Schrumpf, Marcia Gasis, Susanne Grether-Beck, Jean Krutmann, Bernhard Homey, Albert Zlotnik

**Affiliations:** 1 Department of Physiology and Biophysics, University of California Irvine, Irvine, California, United States of America; 2 Department of Dermatology, University of Düsseldorf, Düsseldorf, Germany; 3 Institut für Umweltmedizinische Forschung, Leibniz Research Institute for Environmental Medicine, Düsseldorf, Germany; Università degli Studi di Milano, Italy

## Abstract

Through bioinformatics analyses of a human gene expression database representing 105 different tissues and cell types, we identified 687 skin-associated genes that are selectively and highly expressed in human skin. Over 50 of these represent uncharacterized genes not previously associated with skin and include a subset that encode novel secreted and plasma membrane proteins. The high levels of skin-associated expression for eight of these novel therapeutic target genes were confirmed by semi-quantitative real time PCR, western blot and immunohistochemical analyses of normal skin and skin-derived cell lines. Four of these are expressed specifically by epidermal keratinocytes; two that encode G-protein-coupled receptors (GPR87 and GPR115), and two that encode secreted proteins (WFDC5 and SERPINB7). Further analyses using cytokine-activated and terminally differentiated human primary keratinocytes or a panel of common inflammatory, autoimmune or malignant skin diseases revealed distinct patterns of regulation as well as disease associations that point to important roles in cutaneous homeostasis and disease. Some of these novel uncharacterized skin genes may represent potential biomarkers or drug targets for the development of future diagnostics or therapeutics.

## Introduction

DNA microarray technology has been used in numerous expression profiling experiments on mammalian skin designed to understand normal and pathological skin biology but also to characterize the various cell types present in skin at the molecular level [1], [2], [3], [4], [5], [6]. However, there have been no comparative studies on gene expression in normal human skin relative to other tissues. As a consequence, skin-associated genes are poorly represented in sequence databases [5].

We have used DNA microarray technology to measure the expression levels of more than 47,000 unique transcripts in 105 different adult human tissue and cell types. The results provide a comprehensive genome-wide perspective of each tissue’s expression signature. We have called this database the Body Index of human Gene Expression (BIGE) [7], [8]. In the present study, we have used the BIGE database to identify skin-associated genes (SAGs). To this end, we compared the expression value of a given gene in skin to its mean relative expression value in all other tissues and cell types (n = 104) and selected genes that are highly expressed in the skin and little or no expression elsewhere. The resulting set of 687 genes was classified into functional classes utilizing both manual and automated annotation to better understand the molecular composition of skin and to identify significant pathways. Furthermore, we have focused on the top 100 genes preferentially expressed in normal human skin for further characterization. These include a subset of eight poorly characterized genes that are selectively and strongly expressed in human skin which have not been previously associated with skin and encode either secreted or membrane proteins. The skin expression of these novel SAGs was confirmed by semi-quantitative PCR (qPCR) and expression of the proteins encoded by a subset of SAGs was confirmed by immunohistochemistry (IHC) and Western blot. In addition, we analyzed the expression of these genes in cultured primary human keratinocytes and a wide panel of inflammatory, autoimmune or malignant skin diseases and found several important associations. Our results indicate that we have identified several novel skin-associated genes that may represent potential biomarkers and/or drug targets.

## Results and Discussion

### Identification of Skin-associated Genes

We used the BIGE database [7], [8] to identify genes preferentially expressed in human skin. The five skin samples were from two sources; three were collected from the lumbar region of individual donors (two female, one male) and two were total skin RNAs from commercial sources. Selection of genes with a mean skin to mean body (the remaining 104 tissue/cell types) expression ratio ≥2.0 fold resulted in a list of SAGs containing 687 genes (see Table S1).

This systematic approach leverages two powerful attributes of the database: the inclusion of gene expression data from a large spectrum of tissues and cell types, and the use of genome-wide microarray platform (the Affymetrix human genome U133 plus 2.0 array) that provides a comprehensive, “global” profile of gene expression in each sample [7]. By including 104 tissue and cell types (listed in Table S2), our analysis excluded broadly expressed “housekeeping” genes and focused instead on genes that participate in functions and pathways restricted to skin. The use of genome-wide arrays provided data for genes not included in many prior studies, including ∼20% human genes that encode proteins with unknown function [9]. Importantly, a subset of SAGs identified in this study represents uncharacterized genes, as described below.

### Functional Overview

**Figure 1:**
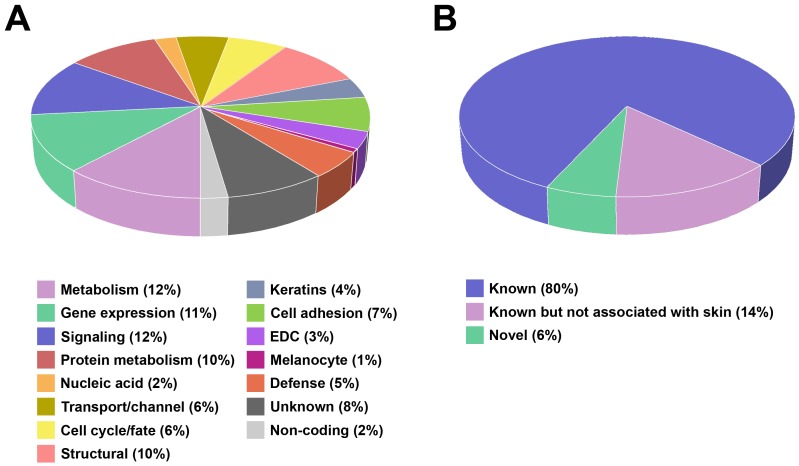
Annotation of the 687 skin-associated genes (SAGs). A) Functional classification. EDC; epidermal differentiation complex genes (see text). Categories with multiple functional classes: Metabolism (Amino acid metabolism, Enzyme, Lipid metabolism, Membrane regeneration/repair, Metabolism), Gene expression (Translation, Transcription factors), Signaling (Receptor, Signaling), Protein metabolism/trafficking (Chaperone, Protease/protease inhibitor, Protein kinase/phosphatase, Protein modification, Protein trafficking, Protein turnover), Nucleic acid binding/metabolism (DNA binding/metabolism, RNA binding/metabolism), Transport/channel (Channel, Transporter, Vesicular trafficking), Cell cycle/fate (Angiogenesis, Apoptosis, Cell cycle, Differentiation, Growth factor/growth factor binding protein, Hormone/hormone binding), Structural (Cytoskeleton, Structural), Cell adhesion (Cell adhesion, Gap junction, Tight junction, Extracellular matrix), Defense (Chemokine/chemokine receptor, Coaggulation, Cytokine/cytokine receptor, Immune response, Innate immunity+Cidal). B) Classification based on level of characterization of top 300 SAGs. See Table S1 for functional annotation for all 687 SAGs.

To understand the biological significance of the full list of 687 SAGs we used manual annotation that utilized gene ontology (GO) term descriptors, published data and protein family (Pfam) sequence alignments to assign SAGs to broad functional classes, and the “Database for annotation, visualization and integrated discovery” (DAVID) tool that systematically identifies functional classes enriched in gene lists [10]. Results from both analyses are summarized in Figure 1A, (see Table S3 for the complete DAVID output).

Metabolism, signaling, gene expression and protein metabolism/trafficking constitute the largest gene classes by manual annotation, representing 35% of our SAGs, indicating that normal skin is a highly active tissue. Many of the remaining functional classes reflect the barrier function of the skin. Our data and mining strategy were validated by the identification of large numbers of genes in the structural, cell adhesion and keratins functional groups and the highly significant enrichment of pathways associated with ectoderm and epidermis development apparent in the DAVID output. Many cell adhesion genes encode desmosome and tight junction components [11] associated with cytoskeletal filaments that form the keratinized squamous cell layer of the skin. We also detected a large number of genes (n = 21) clustered in the epidermal differentiation complex (EDC) locus (located in human chromosomal band 1q21) that encode components of the cornified cell envelope, intermediate filament-associated proteins and calcium binding proteins [12], [13] (see Table S4) also associated with barrier function, and some of which have been implicated in several common skin disorders [14].

Expression of many known SAGs is restricted to hair follicles. Previously, other groups have studied gene expression in hair follicles using laser capture microscopy (LCM), but did not use genome-wide microarrays [15], [16]. 133 of the 687 SAGs we identified were described by Ohyama *et al*. as expressed in hair follicle bulge cells (see Table S5). The unusually high number of genes encoding enzymes involved in lipid metabolism (38 of 82 in the metabolism category, Figure 1A) may be explained by the presence of sebaceous glands, which secrete lipid-rich sebum. Several of the genes we identified, including *SCGB2A2* (rank 3) and *CST6* (cystatin E/M; rank 13), have been localized to the sweat glands [17], [18].

We identified 37 genes associated with skin defenses, including many genes previously linked to both innate or adaptive immune responses as well as several genes encoding antimicrobial peptides (AMPs). Expression of these genes in normal skin suggests the presence of very active mechanisms of microbiome control that operate in the normal skin to prevent bacterial or fungal infections.

### Individual Gene Expression

Table 1 lists the top 100 genes that exhibit the highest specific expression in human skin. The most abundant skin-associated gene, dermcidin (*DCD*), is a well characterized AMP produced by eccrine sweat glands [19]. Interestingly, the *DCD* gene encodes a multifunctional precursor protein, which is cleaved to generate a microbicidal C-terminal 47 amino acid peptide and an N-terminal peptide that acts as a pleiotropic soluble factor associated with cell survival and cachexia [20]. The second most abundantly expressed gene in skin, keratin 2 (*KRT2*, formerly *KRT1B*), is the highest expressed of eleven keratin genes in the top 100 list. Transcripts for the recently described filaggrin family member 2 (FLG2) [21] and desmocollin 1 (*DSC1*), a desmosomal cadherin, are also highly enriched in skin. Both of these proteins participate in skin barrier formation [21], [22]. Two members of the secretoglobin gene superfamily, *SCGB2A2*, (also known as mammaglobin 1) and *SCGB1D2*, (also known as lipophilin B) are also highly expressed in skin. Expression of both secretoglobins in mammary gland has been reported [17], [23], [24], [25], [26], [27]. *SCGB2A2* has also been shown to be expressed in human sweat glands and down-regulated in sweat gland tumors [17]. While the normal function of secretoglobins in healthy tissues remains unclear, other members of this superfamily are anti-inflammatory [28], [29], [30]. Significantly, SCGB1D2 is also present at high levels in tears [31], [32] suggesting a protective role in either defense or barrier function as a mucin-like protein [31]. Both *SCGB2A2* and *DCD* exhibit similar BIGE expression profiles with high expression levels in skin, but relatively low expression levels elsewhere (in contrast to other highly expressed SAGs). As expression of both genes is restricted to eccrine sweat glands, these data may point toward significant differences in the presence and makeup of sweat glands at different sites in the human body with wide distribution of eccrine sweat glands versus limited, site-specific distribution of apocrine glands.

**Table 1:** Top 100 skin-associated genes (SAGs) ranked by fold change of expression compared to the mean of the remaining 104 adult human tissue and cell types in the BIGE.

RANK	GENE TITLE	GENE SYMBOL	RATIO	FUNCTION	CLASS
1	dermcidin	DCD	55.00	Microbicidal	k/k
2	keratin 2	KRT2	31.15	Type II epithelial keratin	k/k
3	secretoglobin, family 2A, member 2	SCGB2A2	30.61	Unknown	k/k
4	filaggrin family member 2	FLG2	29.55	Structural	k/k
5	desmocollin 1	DSC1	21.88	Structural	k/k
6	filaggrin	FLG	18.61	Cytoskeleton	k/k
7	keratin 77	KRT77	18.53	Type II epithelial keratin	k/k
8	late cornified envelope 2B	LCE2B	17.87	Structural	k/k
9	loricrin	LOR	17.65	Structural	k/k
10	late cornified envelope 1B	LCE1B	16.28	Structural	k/k
11	desmoglein 1	DSG1	14.77	Tight junction	k/k
12	dermokine	DMKN	14.37	Signaling	k/k
13	cystatin E/M	CST6	14.17	Protease/protease inh.	k/k
14	desmocollin 3	DSC3	12.82	Tight junction	k/k
15	keratin 1 (epidermolytic hyperkeratosis)	KRT1	12.38	Type II epithelial keratin	k/k
16	mucin-like 1	MUCL1	12.35	Microbicidal	k/n
17	lectin, galactoside-binding, soluble, 7 (galectin 7)	LGALS7	12.07	Immune response	k/k
18	keratin 10	KRT10	12.06	Type I epithelial keratin	k/k
19	secretoglobin, family 1D, member 2	SCGB1D2	11.97	Unknown	k/n
20	keratinocyte differentiation-associated protein	KRTDAP	11.18	GF/GF-binding protein	k/k
21	dopachrome tautomerase (dopachrome delta-isomerase, tyrosine-related protein 2)	DCT	10.38	Melanin BS/melanosome	k/k
22	WAP four-disulfide core domain 5	WFDC5	10.36	Unknown	n/n
23	calmodulin-like 5	CALML5	9.81	Structural	k/k
24	keratin 14	KRT14	9.73	Type I epithelial keratin	k/k
25	chemokine (C-C motif) ligand 27	CCL27	9.65	CK/CK receptor	k/k
26	POU domain, class 2, transcription factor 3	POU2F3	9.26	Transcription factor	k/k
27	plakophilin 1 (ectodermal dysplasia/skin fragility syndrome)	PKP1	9.12	Cytoskeleton	k/k
28	aspartic peptidase, retroviral-like 1	ASPRV1	9.12	Protease/protease inh.	k/k
29	suprabasin	SBSN	8.97	Structural	k/k
30	zinc finger protein 750	ZNF750	8.74	Transcription factor	k/k
31	tyrosinase-related protein 1	TYRP1	8.74	Melanin BS/melanosome	k/k
32	transmembrane protein 45A	TMEM45A	8.72	Unknown	n/n
33	interleukin 1 family, member 7 (zeta)	IL37	8.62	Cytokine/cytokine receptor	k/n
34	pentaxin-related gene, rapidly induced by IL-1 beta	PTX3	8.44	Immune response	k/k
35	GATA binding protein 3	GATA3	8.38	Transcription factor	k/k
36	sciellin	SCEL	8.01	Structural	k/k
37	dystonin	DST	7.74	Tight junction	k/k
38	exophilin 5	EXPH5	7.62	Vesicular trafficking	k/n
39	G protein-coupled receptor 115	GPR115	7.54	Receptor	k/n
40	epiplakin 1	EPPK1	7.49	Structural	k/k
41	cadherin-related family member 1	CDHR1	7.43	Cell adhesion	k/n
42	premature ovarian failure, 1B	POF1B	7.35	Structural	k/k
43	kallikrein 5	KLK5	7.34	Protease/protease inh.	k/k
44	arylacetamide deacety lase-like 2	AADACL2	7.32	Enzyme^*^	n/n
45	stratifin	SFN	7.26	Signaling	k/k
46	desmoplakin	DSP	7.23	Tight junction	k/k
47	claudin 1	CLDN1	7.19	Tight junction	k/k
48	lymphocyte antigen 6 complex, locus D	LY6D	7.12	Tight junction	k/k
49	trichohyalin	TCHH	7.09	Cytoskeleton	k/k
50	keratin 80	KRT80	6.84	Type II epithelial keratin	k/k
51	PERP, TP53 apoptosis effector	PERP	6.83	Tight junction	k/k
52	serine (or cysteine) proteinase inhibitor, clade B (ovalbumin), member 7	SERPINB7	6.68	Protease/protease inh.	k/k
53	tripartite motif-containing 29	TRIM29	6.65	Transcription factor	k/k
54	family with sequence similarity 83, member B	FAM83B	6.50	Unknown	n/n
55	interleukin 20 receptor beta	IL20RB	6.48	Cytokine/cytokine receptor	k/k
56	corneodesmosin	CDSN	6.42	Structural	k/k
57	family with sequence similarity 83, member C	FAM83C	6.35	Unknown	n/n
58	serine (or cysteine) proteinase inhibitor, clade B (ovalbumin), member 5	SERPINB5	6.29	Protease/protease inh.	k/k
59	keratin 5	KRT5	6.29	Type II epithelial keratin	k/k
60	Kruppel-like factor 5 (intestinal)	KLF5	6.26	Transcription factor	k/k
61	transcription factor CP2-like 2	GRHL1	6.24	Transcription factor	k/k
62	tumor necrosis factor receptor superfamily, member 19	TNFRSF19	6.20	Cytokine/cytokine receptor	k/k
63	serine (or cysteine) proteinase inhibitor, clade A (alpha-1 antiproteinase, antitrypsin), member 12	SERPINA12	6.18	Protease/protease inh.	k/k
64	Spectrin, beta, non-erythrocytic 2	SPTBN2	6.15	Protein trafficking	k/n
65	transcription factor AP-2 alpha (activating enhancer binding protein 2 alpha)	TFAP2A	6.11	Transcription factor	k/k
66	SNF1-like kinase	SIK1	6.09	Protein modification	k/n
67	SH3 domain containing ring finger 2	SH3RF2	5.99	Signaling	k/n
68	LY6/PLAUR domain containing 3	LYPD3	5.96	Cell adhesion	k/k
69	lectin, galactoside-binding-like	LGALSL	5.88	Unknown	n/n
70	AHNAK nucleoprotein 2	AHNAK2	5.76	Structural	k/n
71	tenomodulin	TNMD	5.66	Metabolism	k/n
72	giant axonal neuropathy (gigaxonin)	GAN	5.64	Protein turnover	k/k
73	chromosome 5 open reading frame 46	C5orf46	5.64	Unknown	n/n
74	keratin associated protein 2-2	KRTAP2-2	5.63	KRTAP	k/k
75	tumor protein p73-like	TP63	5.58	Transcription factor	k/k
76	keratin 15	KRT15	5.56	Type I epithelial keratin	k/k
77	ATPase, H+ transporting, lysosomal 42kDa, V1 subunit C isoform 2	ATP6V1C2	5.52	Transporter	k/n
78	Xg blood group (pseudoautosomal boundary-divided on the X chromosome)	XG	5.49	Unknown	k/n
79	coagulation factor II (thrombin) receptor-like 1	F2RL1	5.42	Coaggulation	k/k
80	Ly6/neurotoxin 1	LYNX1	5.40	Signaling	k/k
81	keratin 23 (histone deacetylase inducible)	KRT23	5.38	Signaling	k/n
82	forkhead box N1	FOXN1	5.36	Transcription factor	k/k
83	epidermal retinal dehydrogenase 2	SDR16C5	5.35	Enzyme	k/k
84	chloride channel, calcium activated, family member 2	CLCA2	5.35	Channel	k/k
85	BCL2/adenovirus E1B 19kD interacting protein like	BNIPL	5.30	Apoptosis	k/n
86	secreted LY6/PLAUR domain containing 1	SLURP1	5.28	GF/GF-binding protein	k/k
87	annexin A8	ANXA8	5.22	Signaling	k/k
88	keratin 17	KRT17	5.22	Type I epithelial keratin	k/k
89	transcription factor AP-2 gamma (activating enhancer binding protein 2 gamma)	TFAP2C	5.21	Transcription factor	k/k
90	death associated protein-like 1	DAPL1	5.21	Apoptosis	k/k
91	degenerative spermatocyte homolog 1, lipid desaturase (Drosophila)	DEGS1	5.17	Lipid metabolism	k/n
92	syndecan 1	SDC1	5.09	Tight junction	k/k
93	cysteine-rich C-terminal 1	CRCT1	5.05	Structural	k/k
94	chromosome 1 open reading frame 68	C1orf68	5.04	Unknown	k/k
95	epithelial splicing regulatory protein 1	ESRP1	5.03	RNA binding/metabolism	k/k
96	intermediate filament family orphan 2	IFFO2	4.99	Cytoskeleton^*^	n/n
97	tuftelin 1	TUFT1	4.97	Cell cycle	k/k
98	chromosome 19 open reading frame 33	C19orf33	4.92	Apoptosis	k/n
99	keratin 25	KRT25	4.88	Type I epithelial keratin	k/k
100	S100 calcium binding protein A2	S100A2	4.86	Signaling	k/k

**Figure 2:**
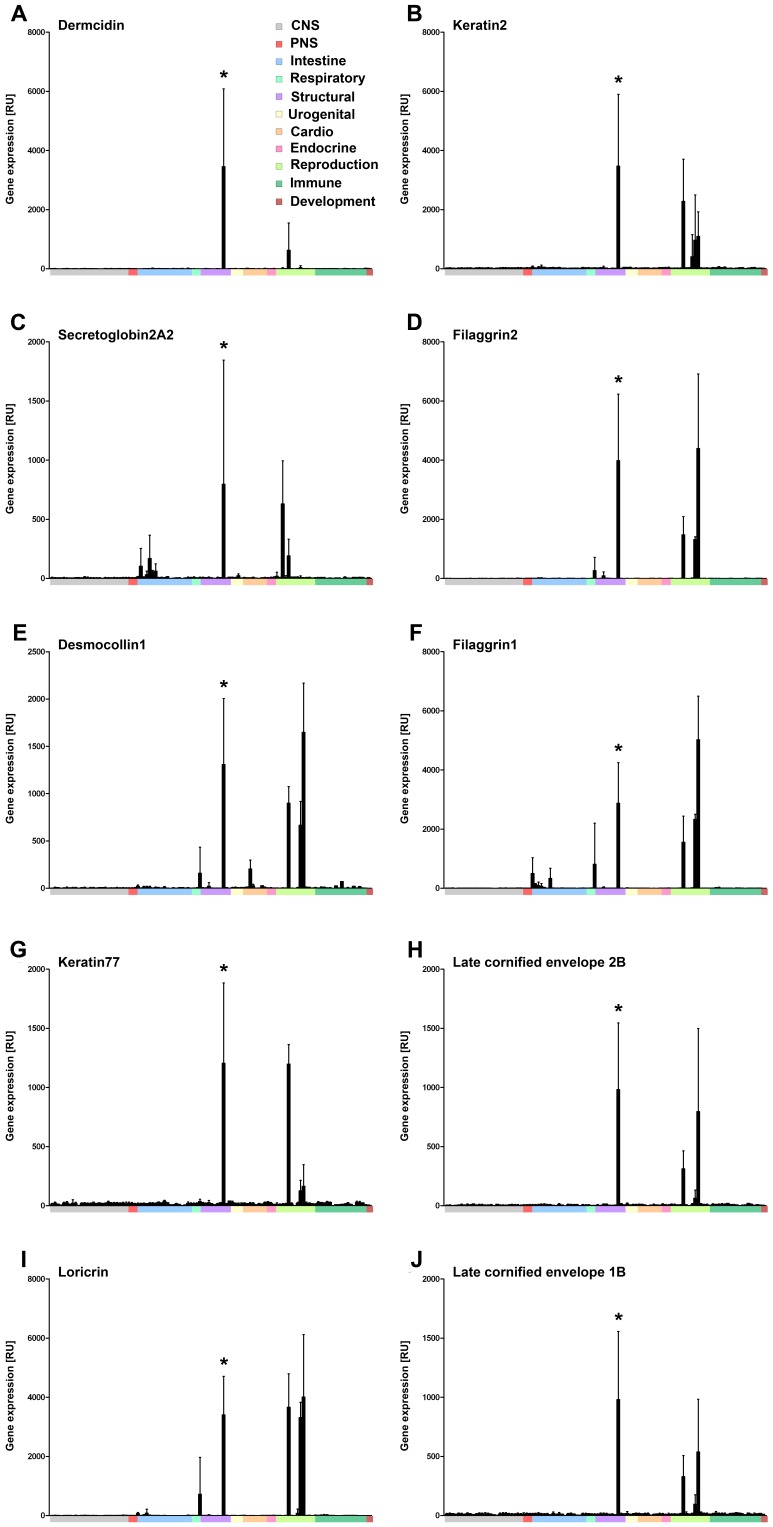
Expression profiles of top human skin-associated genes in the body index of gene expression (BIGE). Affymetrix GeneChip data for the top 10 SAGs are shown as mean normalized relative expression (RU) (+) Standard deviation (y axis), plotted against the sample IDs from 105 human tissue and cell types grouped by system (x axis), as listed in Panel A. CNS, central nervous system; PNS, peripheral nervous system. Asterisk marks skin sample in each profile.

Taken together, our data indicate that nine out of the top 10 genes we have identified qualify as highly specific skin-markers, showing exclusive expression in skin or in other skin-containing organs (i.e. nipple and penis). *SCGB2A2* is an exception, given it is also expressed in the mammary gland (Figure 2 and see Table S7).

Some immune system-associated genes are highly expressed in normal skin. Indeed, sixteen genes encoding cytokines, chemokines and their receptors are present in the full list of 687 SAGs including four in the top 100: *CCL27, IL-1F7/IL-37, IL-20RB* and *TNFRSF19*. *CCL27*, the highest expressed immune associated gene we identified, is a skin-associated chemokine expressed by epidermal basal keratinocytes, which chemoattracts skin-homing cutaneous lymphocyte-associated antigen bearing (CLA^+^) T cells [33] and has been associated with allergic and irritant reactions [34] as well as atopic dermatitis [35]. Another homeostatic chemokine, CXCL14 (rank 154), is also highly expressed in normal skin and other mucosal sites [36], [37] and exhibits direct microbicidal activity [38], [39] suggesting that it may play an active role in the control of the skin microbiome. Two members of the IL-1 family, *IL-37* and *IL-36G*, are prominently expressed in skin and may play antagonistic roles in skin immunity. Pro-inflammatory IL-36G induces anti-microbial peptide expression by keratinocytes in psoriasis [40], whereas, IL-37 acts as a repressor of innate immunity [41], [42]. The balance of these two cytokines may contribute to the control of inflammation in the skin.

IL-37 represents the second most skin-specific cytokine, after CCL27 (see Figure S2 and Table S7). The *IL-37* gene appears late in evolution and does not exist in the mouse. The latter observation may explain why it has not been previously associated with the skin. Results from a recent study linking *IL-37* and psoriatic arthritis suggest that this gene may also play a role in autoimmunity [43].

According to information currently available in PubMed and GenBank, 20 of the top 100 SAGs identified in our study have not previously been associated with the skin. Of these, 14 represent known genes while 6 represent uncharacterized transcripts (Figure 1B). A similar trend was observed for the top 300 genes (see Figure S1 and Table S6).

Notably, our complete analysis identified >50 genes with no informative annotation or functional association. This represents one of the most significant collections of novel skin-associated genes. Given that only about 18% of previous microarray studies on skin gene expression used genome-wide arrays and none utilized gene expression in other tissues as a filter may explain these results (http://www.ncbi.nlm.nih.gov/gds/). Of greatest interest are the poorly characterized genes in the top 300 list that encode highly expressed secreted or transmembrane proteins as these represent potential targets for diagnosis and/or treatment of skin disease (see Table S6). We therefore undertook a more detailed expression analysis of eight of these genes (see Table S8).

### Selected Novel Skin Associated Genes

All eight selected novel SAGs are expressed at high levels in human skin, with expression ratios ranging between 4.6 for *GPR87* to 12.4 for *MUCL1*, (see Figure S2 and Table S7). Four of these genes – *MUCL1* (rank 16), *WFDC5* (rank 22), *SERPINB7* (rank 52) and *C5orf46* (rank 73) – encode small predicted secreted proteins while the remaining four – *TMEM45A* (rank 32), *GPR115* (rank 39), *CDHR1* (rank 41) and *GPR87* (rank 110) – encode integral plasma membrane proteins.

**Figure 3:**
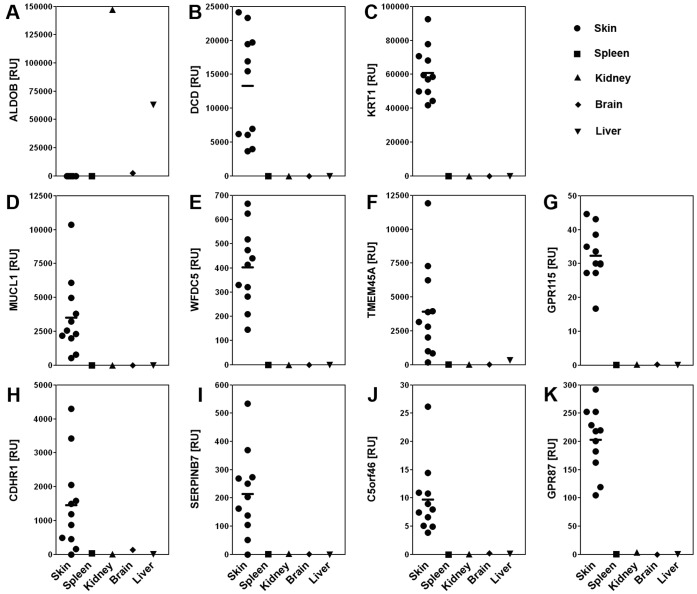
Semi-quantitative PCR confirmation of the specificity of expression of poorly characterized skin-associated genes (SAGs) in human total RNAs. Expression levels of three control genes: (A) ALDOB, aldolase B fructose-biphosphate (not expressed in skin); (B) DCD, dermcidin; and (C) KRT1, keratin 1; and eight selected SAGs: (D) MUCL1, mucin-like 1; (E) WFDC5, WAP four-disulfide core domain 5; (F) TMEM45A, transmembrane protein 45A; (G) GPR115, G protein-coupled receptor 115; (H) CDHR1, cadherin-related family member 1; (I) SERPINB7, serpin peptidase inhibitor, clade B (ovalbumin), member 7; (J) C5orf46, chromosome 5 open reading frame 46; (K) GPR87, G protein-coupled receptor 87, were measured in healthy skin (eleven independent samples) and pooled total RNAs from spleen, kidney, brain and liver measured relative to control gene levels (18S RNA) and plotted as individual ratios, including, for skin samples, mean ratio values shown by the horizontal bar.

We confirmed high levels of expression in normal skin for all eight uncharacterized and two established SAGs (*KRT1*; rank 15, and *DCD*; rank 1) using qPCR (Figure 3) in eleven independent normal human skin samples. These SAGs exhibited little or no expression in other human tissue samples tested. Measurement of the expression of aldolase B (*ALDOB*), which is expressed by kidney and liver, confirmed our analysis showing a marked expression in these organs but no expression in spleen, brain or skin [44].

**Figure 4:**
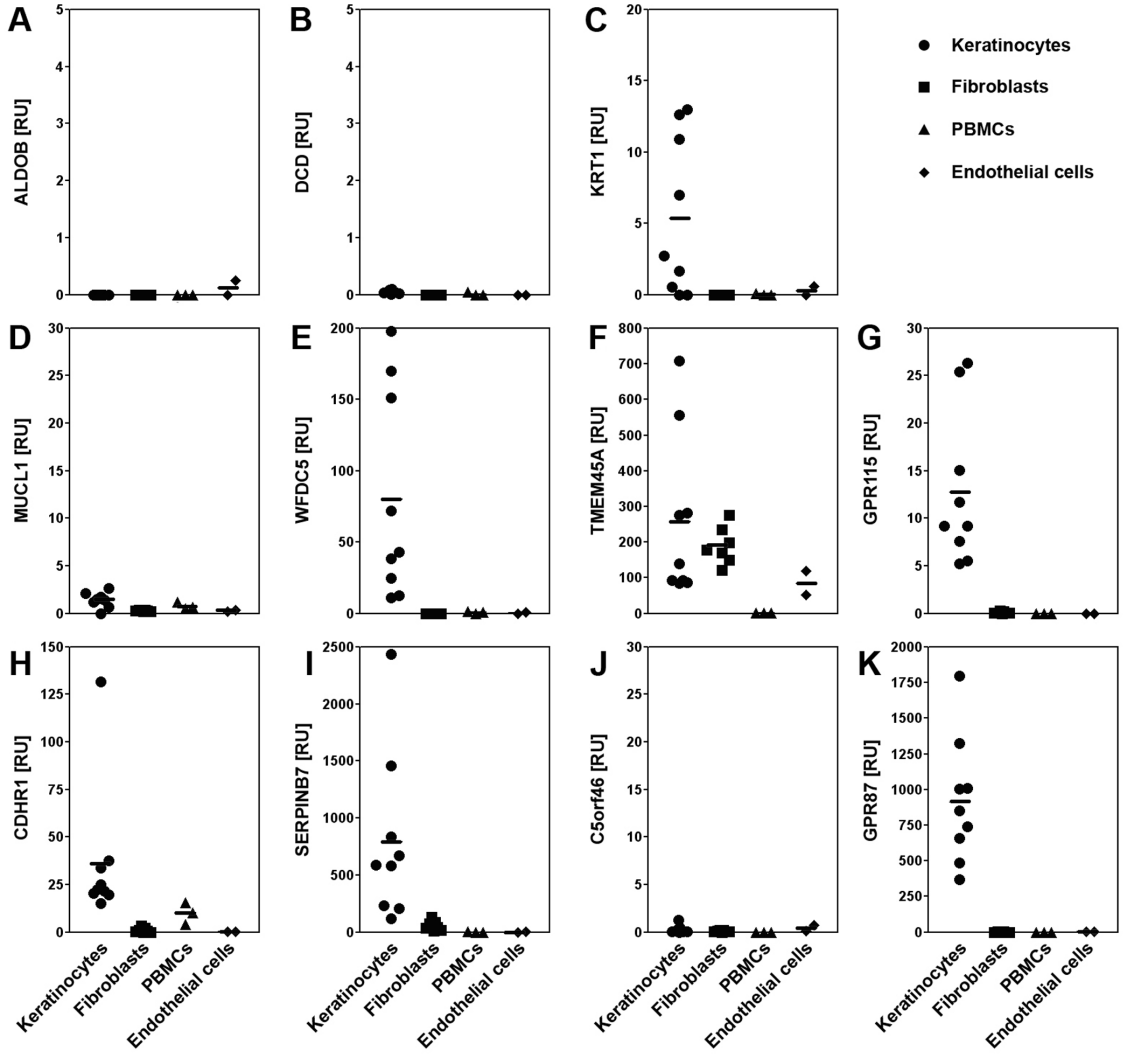
Expression of poorly characterized skin-associated genes in human skin-derived cell lines. Semi-quantitative PCR analysis of expression of three control genes: (A) ALDOB, aldolase B fructose-biphosphate (not expressed in skin); (B) DCD, dermcidin; and (C) KRT1, keratin 1; and eight selected SAGs: (D) MUCL1, mucin-like 1; (E) WFDC5, WAP four-disulfide core domain 5; (F) TMEM45A, transmembrane protein 45A; (G) GPR115, G protein-coupled receptor 115; (H) CDHR1, cadherin-related family member 1; (I) SERPINB7, serpin peptidase inhibitor, clade B (ovalbumin), member 7; (J) C5orf46, chromosome 5 open reading frame 46; (K) GPR87, G protein-coupled receptor 87, were measured in low passage number primary human cells relative to control gene levels (18S RNA) and plotted as individual and mean ratios (horizontal bar).

We also used qPCR to identify the cell type(s) that express each selected SAG using cultured primary human skin-derived cells (Figure 4). Four genes (*GPR87, GPR115, SERPINB7* and *WFDC5*) are expressed exclusively by human primary keratinocytes (PHKs) with no detectable expression in the remaining three cell types. *TMEM45A* is expressed in PHKs, fibroblasts and endothelial cells while *CDHR1* is expressed in PHKs and PBMCs. This pattern explains their high expression in skin, as keratinocytes constitute 95% of skin cells. Surprisingly, despite showing marked skin-specific expression, we did not detect expression of *DCD, C5orf46* or *MUCL1* in the skin cells tested. As *DCD* expression is known to be restricted to eccrine sweat glands, these data suggest that both C5orf46 and MUCL1 may also be secreted by eccrine sweat or by seborrheic glands [19].

MUCL1 (also known as SBEM) is a small secreted sialomucin (similar to SCGB1D2 and SCGB2A2) that has been proposed as a breast cancer biomarker [45]. Accordingly, *MUCL1* is significantly expressed in normal mammary gland (a pattern shared by other genes including *keratins 5, 14, 15 and 17*), and also desmoplakin, a tight junction protein [46], [47], [48]. This suggests common features shared between the skin and mammary gland proteomes. Indeed, myoepithelium, which is found in glandular structures present in sweat, mammary, lacrimal and salivary glands, is composed of keratin-rich epithelial cells and may be the source of these commonly expressed transcripts [49]. Interestingly, both the *MUCL1* and *DCD* genes are in the same locus on chromosome 12 (12q13) and adjacent to another small secreted glycoprotein, lacritin, which is expressed in the lacrimal and mammary gland tissue as well as in breast cancer [50], [51].

Cadherin-related family member 1 (*CDHR1*; formerly known as protocadherin 21, *PCDH21*) is a calcium-dependent adhesion protein and belongs to the ε-group of non-clustered protocadherins [52]. *CDHR1* is a proposed photoreceptor-specific gene and *CDHR1* null-mutations have been shown to cause retinitis pigmentosa [52], [53].

WAP four-disulfide core domain 5 (WFDC5) is one of an eighteen-member family of small secreted proteins originally reported as protease inhibitors but now known to also participate in inflammation and host defense [54], [55], [56]. However, the function(s) and sites of expression of *WFDC5* are currently unknown. Our data strongly suggests that *WFDC5* expression plays a role in inflammatory skin diseases and host defense.

TMEM45A is a highly conserved protein with strong sequence conservation in vertebrates (47% identity with its Zebrafish ortholog) and limited identity to OAS1, a protein involved in anti-viral responses [57]. TMEM45A contains the Pfam domain DUF716 (domain of unknown function 716) associated with viral infection in plants (InterPro IPR006904). Thus, this gene and its protein product may play a role in immunity in the skin. A recent study reported that *TMEM45A* is overexpressed in breast cancer and is associated with poor prognosis in these patients [58]. Conversely, *TMEM45A* suppression promotes the progression of ductal carcinoma *in situ* to invasive breast cancer [59].

Human clade B serpins belong to a cohort of evolutionarily dispersed proteinase inhibitor clades that protect cells from promiscuous proteolysis [60]. *SERPINB7* (Megsin) has been shown to be specifically expressed in kidney and in epithelial cells of the uterus [61], [62]. Over expression of human *SERPINB7* in transgenic mice led to increased mesangial cell proliferation and extracellular matrix (ECM) deposition [61] and, in an *in vitro* cell invasion/migration assay, markedly suppressed cell motility and invasion [63]. Thus, this gene product appears to participate in tissue integrity by maintaining ECM homeostasis and loss of expression may lead to loss of cell adhesion and tissue integrity.

GPR115 is one of 33 human adhesion-GPCRs characterized by long extracellular N-terminal domains [64]. It was identified in a systematic search for glucocorticosteroid-response genes and may mediate the effects of this hormone in the skin [65]. GPR87, a rhodopsin-type (class A) GPCR, was identified by sequence data mining [66], [67] and was later shown to be overexpressed in squamous cell carcinoma [68] and act as a pro-survival, p53-induced gene [69]. Taken together, these observations suggest that GPR87 may play a role in skin cancers.

**Figure 5:**
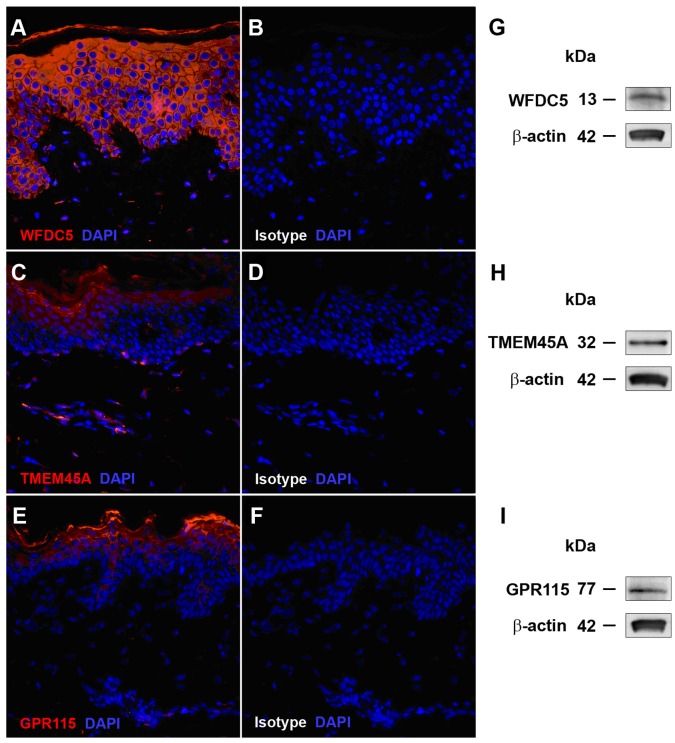
Detection of selected poorly characterized SAG encoded proteins in normal human skin and primary keratinocytes. Panels A–F; formalin-fixed paraffin embedded normal human skin sections were stained for DNA with DAPI and also with either SAG protein-specific (A, C, E), or isotype control antibodies (B, D, F) and visualized by immunofluorescent microscopy, 40× magnification. WFDC5, TMEM45A and GPR115 protein expression compared to beta actin was also detected in primary cultured human keratinocyte lysates using Western blotting (panels G, H and I respectively).

We confirmed the expression of three SAGs (*WFDC5, TMEM45A* and *GPR115*) at the protein level using IHC of both PHKs and healthy skin sections (Figure 5). WFDC5 staining was significant in the cytoplasm of keratinocytes throughout the stratum basale (SB), stratum spinosum (SS) and stratum granulosum (SG) of the epidermis. In the stratum corneum (SC) *WFDC5* expression was confined to the most apical layer. In addition, WFDC5 staining was also apparent in the small vessel endothelia of the dermis possibly due to the protein accumulating on the luminal surface of these vessels. TMEM45A was also expressed in the epidermis and exhibited an increase in intensity from the SB to the SC. As expected, TMEM45 was also detected in dermal fibroblasts. GPR115 shows weak expression in the SB and a marked expression in SG and SC. Increases in expression from basal to apical layers of the epidermis for both TMEM45A and GPR115 suggest that their expression increases upon differentiation. The keratinocyte-specific expression at the protein level was confirmed for all three genes by Western blot.

**Figure 6:**
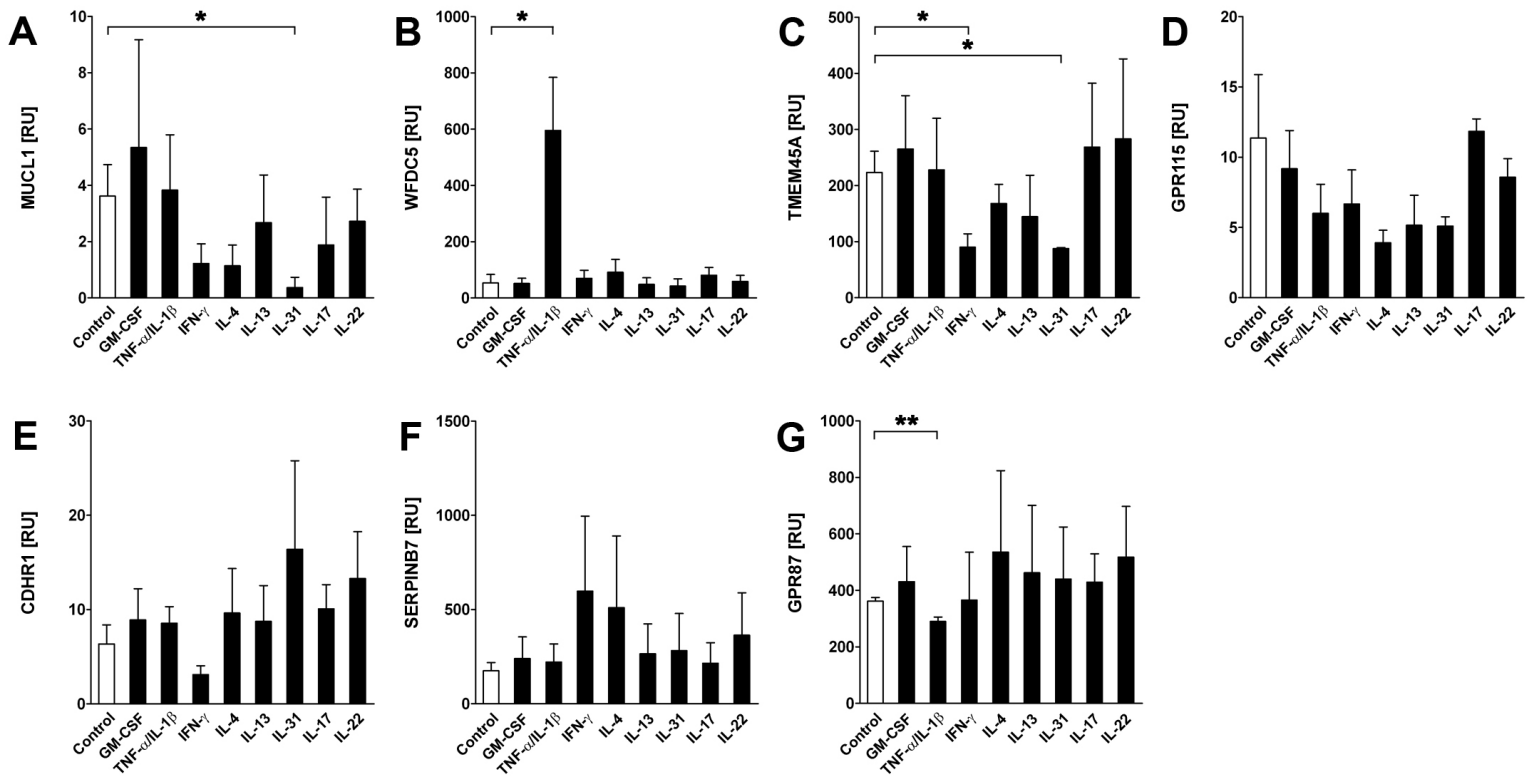
Regulation of poorly characterized skin-associated gene expression in primary human keratinocytes. Semi-quantitative PCR analysis of expression of seven of the eight selected SAGs in primary human keratinocytes incubated with either cytokines (as indicated) or medium controls for 24 hours relative (RU) to control gene levels (18S RNA). Data shown are the mean ± SD. *p<0.05, **p<0.01 (Student’s t-test). SAGs tested were: (A) MUCL1, mucin-like 1; (B) WFDC5, WAP four-disulfide core domain 5; (C) TMEM45A, transmembrane protein 45A; (D) GPR115, G protein-coupled receptor 115; (E) CDHR1, cadherin-related family member 1; (F) SERPINB7, serpin peptidase inhibitor, clade B (ovalbumin), member 7; (G) GPR87, G protein-coupled receptor 87. C5orf46 expression was not detected in keratinocytes.

**Figure 7:**
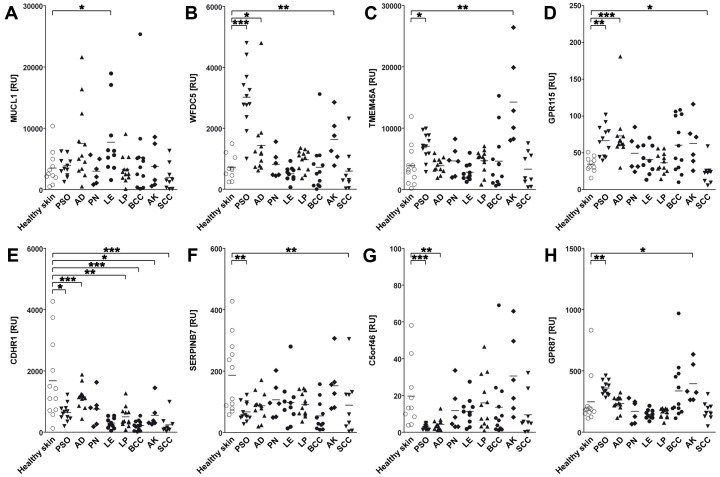
Expression of poorly characterized skin-associated genes in human healthy skin vs. skin diseases. PSO, Psoriasis; AD, Atopic dermatitis; PN, Prurigo nodularis; LE, Lupus erythematosus; LP, Lichen planus; BCC, Basal cell carcinoma; AK, Actinic keratosis; SCC, Squamous cell carcinoma. Semi-quantitative PCR analysis of expression of eight selected SAGs; A–H, in healthy or diseased human skin biopsies relative to control gene levels (18S RNA), plotted as individual and mean ratios (horizontal bar). *p<0.05; **p<0.01; ***p<0.001 (Mann-Whitney-U test).

Next, we sought to analyze the functional regulation of our selected SAGs in primary human keratinocytes and also in common human skin diseases. As *C5orf46* did not show expression in primary keratinocytes and is potentially expressed by another skin-resident cell subset (Figure 4), this gene was excluded from the keratinocyte analyses. Primary human keratinocytes (PHKs) were incubated with medium controls or a panel of cytokines, including GM-CSF, TNF-α/IL1-β, IFN-γ, IL-4, IL-13, IL-31, IL-17 and IL-22. The relative expression of selected SAGs was subsequently measured by qPCR (Figure 6). The following skin diseases were selected for our survey: psoriasis vulgaris (PSO), atopic dermatitis (AD), prurigo nodularis (PRU), lupus erythematosus (LE), lichen planus (LP), basal cell carcinoma (BCC), actinic keratosis (AK) and squamous cell carcinoma (SCC), (Figure 7). As none of our selected SAGs showed any expression in melanocytic nevi or malignant melanoma, these entities were excluded from our disease panel (data not shown).

We found distinct patterns of regulation for each gene. *MUCL1* expression was significantly inhibited by IL-31 (Figure 6A). IL-31 is a recently discovered T-cell derived cytokine that is overexpressed in pruritic skin inflammation [70]. In contrast to the *in vitro* results, *MUCL1* was only significantly upregulated in LE and not conditions associated with pruritis (Figure 7A).

*WFDC5* showed a marked and significant induction by TNF-α/IL1-β (Figure 6B). Notably, *WFDC5* was upregulated significantly in PSO, followed by AK and AD (Figure 7B). The latter observation, along with the fact that the WFDC protein family has been proposed to play an important role in inflammation [54], [55], [56], strongly suggests that WFDC5 is a proinflammatory protein. PSO represents one of the major inflammatory skin disorders and TNF-α is one of the predominant cytokines involved in the pathogenesis of this disease. Notably, anti-TNF-α therapy has evolved as an effective strategy in the management of PSO [71], [72]. Moreover, other WFDC proteins have been reported to exhibit antimicrobial activity [54]; this leads us to postulate that WFDC5 may also have antimicrobial activity. This hypothesis is supported by the clinical observation that PSO patients seldom develop cutaneous infections. Furthermore, PSO scales are a good source of skin-derived antimicrobial proteins [73]. We conclude that WFDC5 deserves further study to clarify its potential role in the pathogenesis of PSO.

The expression of *TMEM45A* was significantly suppressed by IFN-γ while *GPR115* expression was impaired by TNF-α/IL1-β, IFN-γ, IL-4, IL-13 and IL-31 (Figures 6 C and D). *GPR87* was markedly induced by IL-4 and IL-22, whereas TNF-α/IL1-β stimulation resulted in significant suppression of expression (Figure 6G). Disease-association analyses revealed a significant upregulation of *TMEM45A* in PSO and in AK (Figure 7C). Similar regulation was observed for both skin-associated GPCRs, *GPR115* and *GPR87* (Figure 7D and G). However, despite their induction in AK (which defines common precursors to sun-related SCC), their expression in SCC was reduced. Interestingly, TMEM45A was recently reported to suppress the progression of ductal carcinoma *in situ* to invasive breast cancer [59]. Additional studies are needed to address whether TMEM45A may play a similar role in the progression from AK to SCC. *GRP87* is induced by the p53 tumor suppressor gene [69] and knockdown of *GPR87* sensitizes breast and colon cancer cells to DNA damage-induced growth suppression in a p53-dependent manner. Our observations suggest that GPR87, GPR115 and/or TMEM45A may have similar functions in skin cancers. Interestingly, *TMEM45A* expression in primary human keratinocytes is induced upon differentiation [58]. These findings are in line with the results of our immunohistochemical analyses, showing increased TMEM45A staining from the basal layers of the epidermis to the stratum corneum (Figure 5c). Similar staining patterns were observed for GPR115 and, to a lesser extent, for WFDC5, indicating that these proteins are induced upon differentiation (Figure 5A and E), with maximal expression in the stratum granulosum. Double-immunofluorescence analyses with filaggrin, a protein known to be expressed in the upper layers of the epidermis, demonstrated co-localization with TMEM45A and GPR115 in the most apical layers of the epidermis and in particular in the stratum corneum (see Figure S3).

To characterize differentiation-dependent regulation in more detail, we followed the expression levels of the three SAGs and four control genes in PHKs induced to terminally differentiate by confluence [74] or by varying Calcium ion levels [75]. qPCR analysis demonstrated induction of expression for all four differentiation marker genes as well as all three novel SAGs upon differentiation (see Figures S4, S5). These findings also correlate in the upregulation of all three genes in PSO, as the histomorphologic characteristics of PSO include excessive hyperkeratosis and acanthosis with abundant keratinocytes and dysregulated differentiation. This relationship is especially significant for *WFDC5* which exhibits the largest increase in expression in both *in vitro* differentiation models and in PSO. The additional significant induction of *GPR115* in AD points to a potential role for this receptor in the pathogenesis of inflammatory skin disorders and may be associated with the therapeutic effects of glucocorticosteroids in these diseases [65].

*CDHR1* expression was suppressed by IFN-γ, whereas we observed a marked induction by IL-31 and IL-22 (Figure 6E). *CDHR1* expression was suppressed in all skin diseases included in our screen, except PRU (Fig. 7E). *CDHR1* is a photoreceptor-specific gene and its expression and function in skin is currently unknown [52], [53]. *CDHR1* expression in healthy skin covers a broad range from nearly zero to >4000 relative units. The physiological significance of this observation is unknown.

The proteinase inhibitor SERPINB7 also exhibits substantial variation of expression in normal skin and while our data reveal significant downregulation in PSO and SCC, and the role of SERPINB7 in these diseases is uncertain. Shiiba et al. demonstrated that *SERPINB7* and other genes of the *SERPIN* gene cluster on chromosome 18q21 were significantly downregulated in cell lines derived from oral SCC [76] and concluded that these *SERPIN* members may represent tumor suppressor genes. Our data are consistent with this hypothesis. Finally, *C5orf46* was downregulated in PSO and AD. Further studies are needed to identify the cellular origin of this protein and to gain further insights into its regulation and potential role in skin disorders.

Our study represents the most comprehensive comparative molecular profiling of normal human skin to date. The overall strategy of our analytical approach was validated by our results: among the top 100 SAGs, we identified 14 known genes not yet known to be expressed in skin, as well as another 6 skin-expressed uncharacterized transcripts. Further analyses of selected SAGs indicate that several of these genes exhibit altered expression in a several human skin diseases. Our observations strongly suggest that some of these genes participate in the pathogenesis of these human skin diseases.

## Materials and Methods

### Tissue Samples

All human biopsy skin samples were collected with written consent from the donors and with the approval of the Medical Faculty of the University of Düsseldorf IRB. All samples received from Zoion Diagnostics were approved by an IRB in previous studies by the authors [7], [8].

### Gene Expression Data Acquisition and Analysis

Generation of the Body Index of Gene Expression database (BIGE) has been described [7]. Briefly, flash-frozen human tissue samples were obtained from multiple male and female human donors, between 3–5 hours post-mortem (Zoion Diagnostics, Hawthorne, NY), (see Table S2). Gene expression data was obtained using Affymetrix Human Genome U133 Plus 2.0 gene arrays and standard protocols (Affymetrix, Santa Clara, CA). Background adjustment, signal normalization, and summarization were done using the Robust Multi-array Average (RMA) algorithm [77] in ArrayAssist software (Iobion Labs, La Jolla, CA). To identify genes expressed exclusively or predominantly in the skin [skin-associated genes (SAGs)], we calculated the ratio between the means of the skin samples (n = 5) and the samples representing the remaining body tissues and cell types (n = 435) and selected genes with skin to body expression ratios ≥2.0.

### Collection of Skin Samples and Cell Culture

Skin biopsies were taken after obtaining informed written consent from healthy individuals undergoing plastic surgery; n = 11, or from lesional skin of individuals with common skin diseases (psoriasis vulgaris; n = 12; atopic dermatitis; n = 12, prurigo nodularis; n = 6, lupus erythematosus; n = 10, lichen planus; n = 12, basal cell carcinoma; n = 12, actinic keratosis; n = 6, squamous cell carcinoma; n = 9. Skin biopsies were immediately snap-frozen in liquid nitrogen and stored at –80°C.

Primary human cells were cultured at 37°C in 5% CO_2_ in cell-specific media supplemented with L-Glutamate (2 mM) and antibiotics (penicillin 100 U/ml, streptomycin 100 µg/ml). For keratinocytes; keratinocyte medium (GIBCO, Invitrogen, Carlsbad, CA) was supplemented with recombinant EGF and bovine pituitary extract. For fibroblasts; fibroblast medium Quantum 333 (PAA, Pasching, Austria), and for endothelial cells; endothelial cell medium EGM MV (Lonza, Basel, Switzerland). Peripheral blood mononuclear cells (PBMCs) were isolated from buffy coats by Ficoll-Paque (GE Healthcare, Pittsburgh, PA) density-gradient centrifugation.

As control samples for human spleen, kidney, brain and liver total RNA were obtained from Clontech, Mountain View, CA.

For functional analyses, cytokines (R&D Systems, Wiesbaden-Nordenstadt, Germany) were used to stimulate primary human keratinocytes using the following concentrations: GM-CSF (50 ng/ml), TNF-α (10 ng/ml)+IL-1β (5 ng/ml), IFN-γ (50 ng/ml), IL-4 (50 ng/ml), IL-13 (100 ng/ml), IL-31 (10 ng/ml), IL-17 (50 ng/ml) or IL-22 (50 ng/ml) for 24 hours. For keratinocyte differentiation, primary human epidermal keratinocytes (NHK) were prepared from neonatal foreskin and maintained in culture under serum-free conditions as described previously [78]. For confluence-induced differentiation, NHK were seeded and grown up to confluence and maintained for 48 hrs to induce differentiation [79]. For calcium-induced differentiation, cells were maintained in low (0.07 mM) or high (1.2 mM) calcium concentrations for 24 hours [80]. Total RNA was isolated and gene expression measured by a two-step reverse transcription real time PCR as described [79]. qPCR results are plotted as ratios relative to the control gene (GAPDH) in universal standard cDNA, see [81].

### Quantitative Real-Time PCR (TaqMan) Analysis

qPCR analysis was performed as described [82]. Primers were obtained from Eurofins MWG, Ebersberg, Germany: ALDOB forward 5′-CAAGGCTGCAAACAAGGAG-3′, reverse 5′-CCCGTGTGAACATACTGTCCT-3′; DCD forward 5′-AGACCCAGGGTTAGCCAGAC-3′, reverse 5′-CTCCGTCTAGGCCTTTTTCC-3′; KRT1 forward 5′-GCCTCCTTCATTGACAAGGT-3′, reverse 5′-GCTCCCATTTTGTTTGCAGT-3′; C5orf46 forward 5′-ACCCCGTGCTACCAGAATGGCT-3′, reverse 5′-TGCCCGAGTCGTCTGGCTTG-3′; CDHR1 forward 5′-CCCTGGATGCCCTGCACAACA-3′, reverse 5′-CGGGAGAGGGTCTCAGCATCT-3′; GPR87 forward 5′-CACCGTATGAGGTGAATGGA-3′, reverse 5′-TGGGTTCAGCATAGGTTATTCC-3′; GPR115 forward 5′-CCCTGCTGGGCTTGGTCGTC-3′, reverse 5′-CCCACCCCCAACCCTACCCC-3′; MUCL1 forward 5′-TCTGCCCAGAATCCGACAACAGC-3′, reverse 5′-GGGCTTCATCATCAGCAGGACCA-3′; SERPINB7 forward 5′-GGGGGAAAATACCTAGGGCTCAACA-3′, reverse 5′-GGCCATTGCAAAATGGAGTGCAGC-3′; TMEM45A forward 5′-TCGGGTCTGGTTGCCTTCTTGGA-3′, reverse 5′-ACGCCATCTGCCAGAACCAGGT-3′; WFDC5 forward 5′-GCCAGATGATGGGCCCTGCC-3′, reverse 5′-GCAGCTGCCCAGCTTCACAGA-3′. Gene-specific PCR products were measured by means of an ABI PRISM 7700 sequence detection system (Applied Biosystems, Foster City, CA). Target gene expression was normalized to the expression of 18S rRNA.

### Immunohistochemistry and Immunofluorescence

Sections from human skin were routinely fixed with formalin and embedded in paraffin. After demasking with Protease XXIV (Sigma-Aldrich, St. Louis, MO) in 20 mM Tris pH 7.9 the skin sections were incubated with antibodies directed against human WFDC5 (mouse polyclonal; Abcam, Cambridge, UK), TMEM45A (rabbit polyclonal; Sigma-Aldrich), GPR115 (rabbit polyclonal; Abcam) and filaggrin (mouse monoclonal; Abcam) or appropriate isotype control antibodies. Primary antibody binding was detected using the secondary antibodies donkey anti-mouse or rabbit IgG AviAlexa 594 (Invitrogen, Carlsbad, CA) and donkey anti-mouse IgG AviAlexa 488 (Invitrogen). Sections were fixed with Fluoromount G. Immunoreactions were detected by use of a microscope (Axiovert 200 M) (Zeiss, Jena, Germany) using Axiovision 4.7 (Zeiss).

### Western Blot Analysis of WFDC5, TMEM45A and GPR115 Expressed in Human Keratinocytes

Human keratinocytes were harvested and resuspended in lysis buffer (50 mM HEPES [pH 7.4], 150 mM NaCl, 1% NP40, 0.1% SDS, 1% Triton X-100 and protease inhibitor cocktail). The lysates were centrifuged at 5,000 g for 5 min to remove cellular debris, resolved by polyacrylamide electrophoresis and analyzed by Western blotting using the appropriate antibodies according to the manufactureŕs protocol. Protein blots were developed using Western blot detection solution and hyperfilm (Amersham ECL detection reagents; GE Healthcare, Pittsburgh, PA).

## Supporting Information

Figure S1 Top 300 SAG informatics classifications.
(TIF)

Figure S2 BIGE profiles of the three control genes and eight selected poorly characterized SAGs described in the text. Affymetrix GeneChip data are shown as mean normalized relative expression (RU) (+) Standard deviation (y axis), plotted against the sample IDs from 105 human tissue and cell types grouped by system (x axis). Systems labeled as follows: ZNS, central nervous system; PNS, peripheral nervous system. Profiles shown are: (A) ALDOB, aldolase B fructose-biphosphate (not expressed in skin); (B) DCD, dermcidin; and (C) KRT1, keratin 1; and eight selected SAGs: (D) MUCL1, mucin-like 1; (E) WFDC5, WAP four-disulfide core domain 5; (F) TMEM45A, transmembrane protein 45A; (G) GPR115, G protein-coupled receptor 115; (H) CDHR1, cadherin-related family member 1; (I) SERPINB7, serpin peptidase inhibitor, clade B (ovalbumin), member 7; (J) C5orf46, chromosome 5 open reading frame 46; (K) GPR87, G protein-coupled receptor 87.
(TIF)

Figure S3 Detection of (A) transmembrane protein 45A (TMEM45A) and (B) G protein-coupled receptor 115 (GPR115) in normal human skin sections co-stained with flagillin (FLG), a stratum granulosum-associated protein. Formalin-fixed paraffin embedded normal human skin sections from the same donor (tissue block) were stained with DAPI+ isotype control antibody to determine background staining and visualize cell nuclei and also with either TMEM45A or GPR115 and FLG-specific antibodies and visualized by immunofluorescent microscopy, 40× magnification. Merging of the TMEM45A or GPR115 images with the FLG image shows co-localization of both novel proteins with the known stratum granulosum-associated protein.
(PPTX)

Figure S4 Expression of known skin differentiation marker genes and selected SAGs increases during keratinocyte differentiation. Semi-quantitative PCR analysis of gene expression (mean ± SD) of four known skin differentiation marker genes (top row) and three selected SAGs (WFDC5, WAP four-disulfide core domain 5; TMEM45A, transmembrane protein 45A; and GPR115, G protein-coupled receptor 115) in a confluence-driven *in vitro* model of keratinocyte differentiation after 24, 48, 72 and 96 hours in culture. *p<0.05 one-way ANOVA, with Tukey’s correction.
(PPTX)

Figure S5 Expression of novel skin-associated genes is activated in a calcium-induced model of keratinocyte differentiation. Semi-quantitative PCR analysis of gene expression of: (A) WFDC5, WAP four-disulfide core domain 5; (B) TMEM45A, transmembrane protein 45A; and (C) GPR115, G protein-coupled receptor 115; in primary human keratinocytes cultured at low Ca2+ (normal keratinocyte medium) and high Ca2+ (normal keratinocyte medium +1.2 mM Ca2+). Presented are the results of one experiment.
(JPG)

Table S1 **List of 687 skin-associated genes with expanded annotation.**
(XLSX)

Table S2 Full list of samples included in the body index of gene expression.
(XLSX)

Table S3 Full output of Database for Annotation, Visualization and Integrated Discovery (DAVID) analysis of the list of 678 SAGs.
(XLSX)

Table S4 List of genes located on human chromosome 1q21, the epidermal differentiation complex (EDC) genes with subsets of EDC genes represented a) on the Affymetrix U133 plus 2.0 array and b) in the list of 678 SAGs.
(XLSX)

Table S5 Genes in the list of 678 SAGs previously identified as expressed in hair follicle by Ohyama M, et al. (2006) J Clin Invest 116: 249–260.
(XLSX)

Table S6 Top 300 skin-associated genes (SAGs) ranked by fold change of expression compared to the mean of the remaining 104 adult human tissue and cell types in the BIGE. Classes: k/k, known skin-associated gene; k/n, known gene not previously associated with skin; n/n, uncharacterized (novel) gene. Functional classification as reported in the literature or, by inference, from bioinformatics analysis, marked with an asterisk.
(XLSX)

Table S7 Top 10 tissue or cell types with highest expression of SAGs discussed in text.
(XLSX)

Table S8 Detailed description of the eight selected novel SAGs featured.
(XLSX)
