# Supplementary figures and images for: Systematic Identification and Characterization of Novel Human Skin-Associated Genes Encoding Membrane and Secreted Proteins
Source: PLoS One. 2013 Jun 20;8(6):e63949. doi: 10.1371/journal.pone.0063949 (PMC3688712; doi:10.1371/journal.pone.0063949)

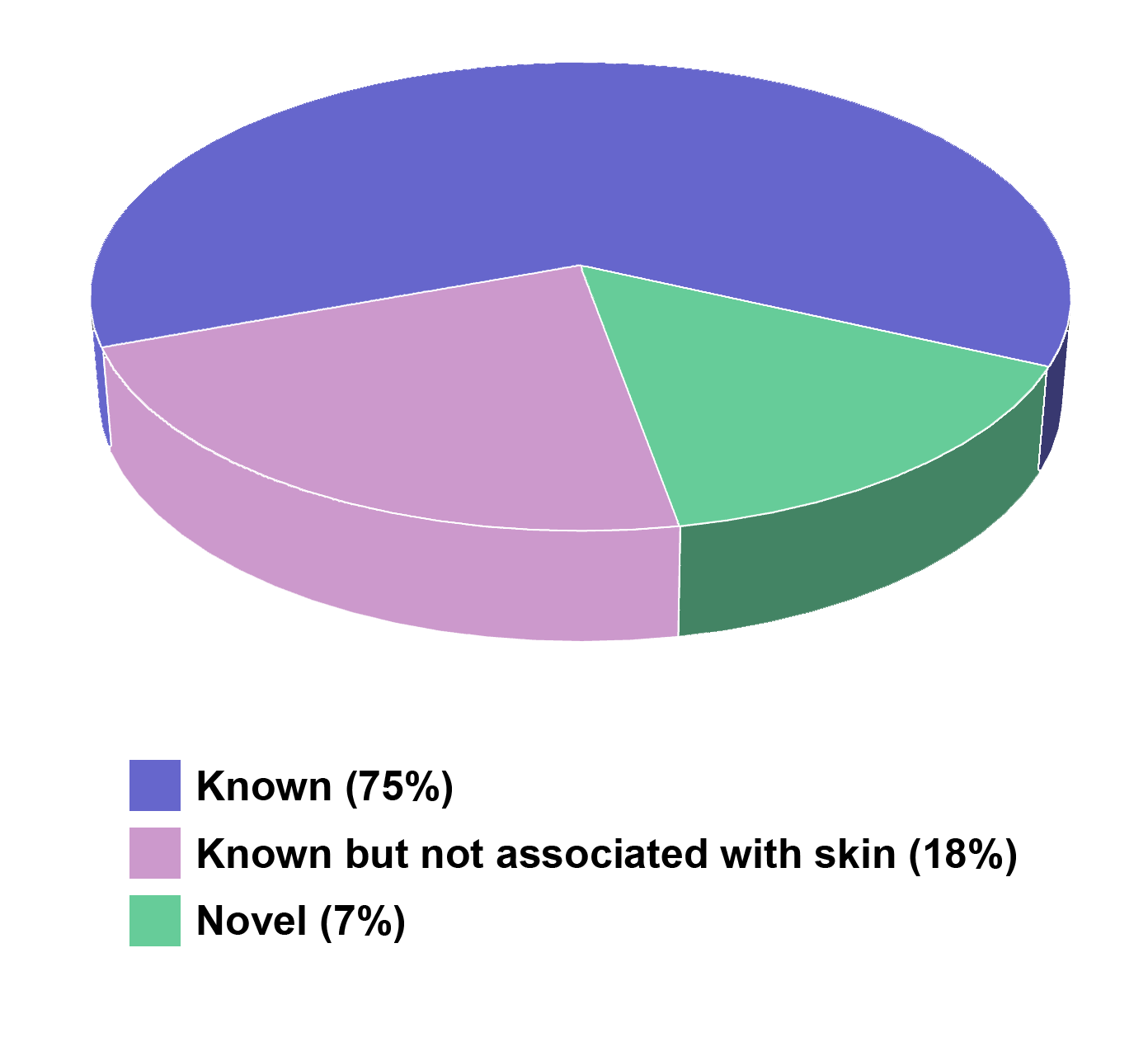

Supplement: Figure S1 — Top 300 SAG informatics classifications. (TIF) [file pone.0063949.s001.tif]

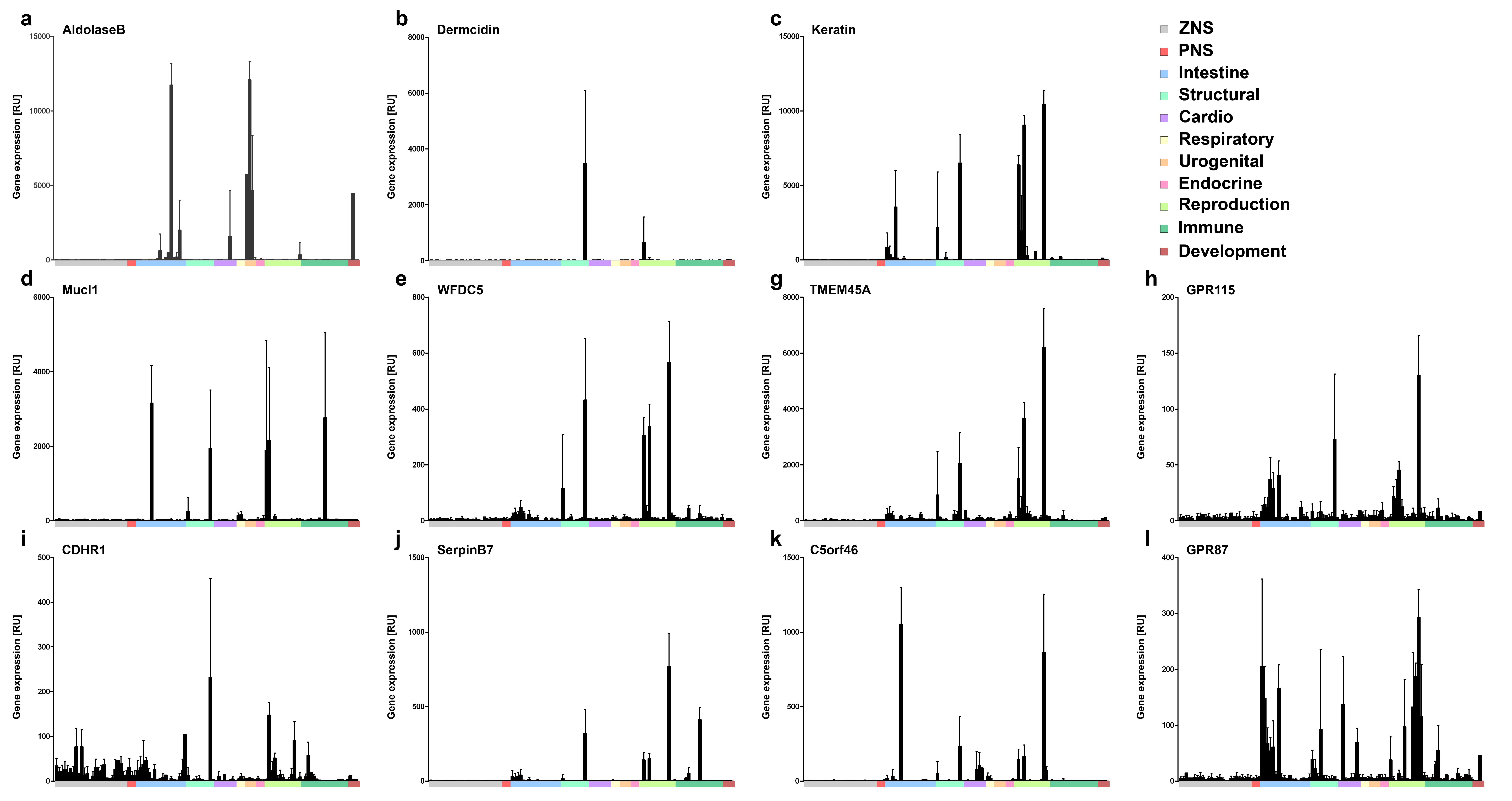

Supplement: Figure S2 — BIGE profiles of the three control genes and eight selected poorly characterized SAGs described in the text. Affymetrix GeneChip data are shown as mean normalized relative expression (RU) (+) Standard deviation (y axis), plotted against the sample IDs from 105 human tissue and cell types grouped by system (x axis). Systems labeled as follows: ZNS, central nervous system; PNS, peripheral nervous system. Profiles shown are: (A) ALDOB, aldolase B fructose-biphosphate (not expressed in skin); (B) DCD, dermcidin; and (C) KRT1, keratin 1; and eight selected SAGs: (D) MUCL1, mucin-like 1; (E) WFDC5, WAP four-disulfide core domain 5; (F) TMEM45A, transmembrane protein 45A; (G) GPR115, G protein-coupled receptor 115; (H) CDHR1, cadherin-related family member 1; (I) SERPINB7, serpin peptidase inhibitor, clade B (ovalbumin), member 7; (J) C5orf46, chromosome 5 open reading frame 46; (K) GPR87, G protein-coupled receptor 87. (TIF) [file pone.0063949.s002.tif]

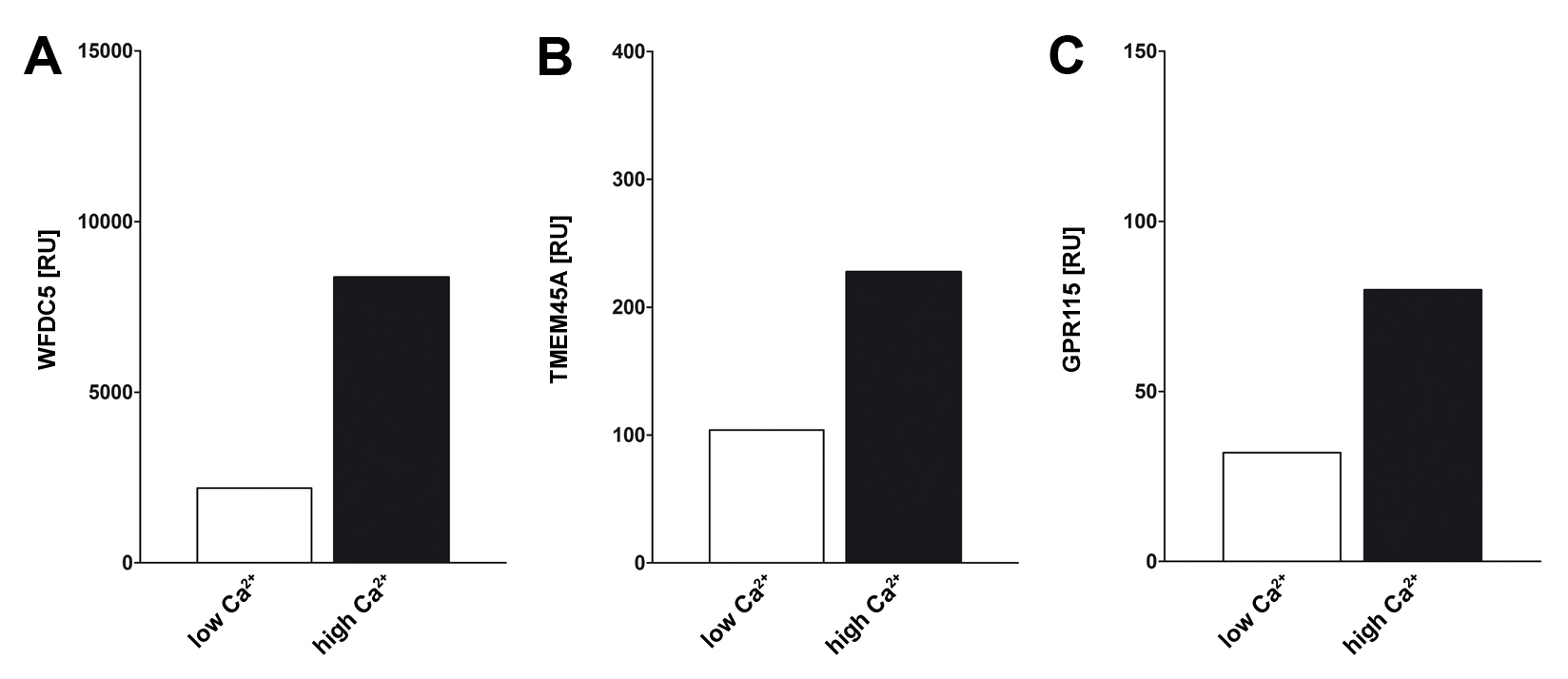

Supplement: Figure S5 — Expression of novel skin-associated genes is activated in a calcium-induced model of keratinocyte differentiation. Semi-quantitative PCR analysis of gene expression of: (A) WFDC5, WAP four-disulfide core domain 5; (B) TMEM45A, transmembrane protein 45A; and (C) GPR115, G protein-coupled receptor 115; in primary human keratinocytes cultured at low Ca2+ (normal keratinocyte medium) and high Ca2+ (normal keratinocyte medium +1.2 mM Ca2+). Presented are the results of one experiment. (JPG) [file pone.0063949.s005.jpg]
